# The Impacts of Albuminuria and Low eGFR on the Risk of Cardiovascular Death, All-Cause Mortality, and Renal Events in Diabetic Patients: Meta-Analysis

**DOI:** 10.1371/journal.pone.0071810

**Published:** 2013-08-30

**Authors:** Tadashi Toyama, Kengo Furuichi, Toshiharu Ninomiya, Miho Shimizu, Akinori Hara, Yasunori Iwata, Shuichi Kaneko, Takashi Wada

**Affiliations:** 1 Division of Nephrology, Kanazawa University Hospital, Kanazawa, Japan; 2 Department of Medicine and Clinical Science, Graduate School of Medical Sciences, Kyushu University, Fukuoka, Japan; 3 Department of Disease Control and Homeostasis, Institute of Medical, Pharmaceutical and Health Sciences, Kanazawa University, Kanazawa, Japan; 4 Department of Laboratory Medicine, Institute of Medical, Pharmaceutical and Health Sciences, Kanazawa University, Kanazawa, Japan; College of Pharmacy, University of Florida, United States of America

## Abstract

**Background:**

Precise effects of albuminuria and low estimated glomerular filtration rate (eGFR) on cardiovascular mortality, all-cause mortality, and renal events in diabetic patients are uncertain.

**Materials and Methods:**

A systematic review was conducted of the literature through MEDLINE, EMBASE, and CINHAL from 1950 to December 2010. Cohort studies of diabetic patients providing adjusted relative risk (RR) of albuminuria and eGFR for risks of cardiovascular mortality, all-cause mortality, and renal events were selected. Two reviewers screened abstracts and full papers of each study using standardized protocol.

**Results:**

We identified 31 studies fulfilling the criteria from 6546 abstracts. With regard to the risk of cardiovascular mortality, microalbuminuria (RR 1.76, 95%CI 1.38–2.25) and macroalbuminuria (RR 2.96 95%CI 2.44–3.60) were significant risk factors compared to normoalbuminuria. The same trends were seen in microalbuminuria (RR 1.60, 95%CI 1.42–1.81), and macroalbuminuria (RR 2.64, 95%CI 2.13–3.27) for the risk of all-cause mortality, and also in microalbuminuria (RR 3.21, 95%CI 2.05–5.02) and macroalbuminuria (RR 11.63, 95%CI 5.68–23.83) for the risk of renal events. The magnitudes of relative risks associated with low eGFR along with albuminuria were almost equal to multiplying each risk rate of low eGFR and albuminuria. No significant factors were found by investigating potential sources of heterogeneity using subgroup analysis.

**Conclusions:**

High albuminuria and low eGFR are relevant risk factors in diabetic patients. Albuminuria and low eGFR may be independent of each other. To evaluate the effects of low eGFR, intervention, or race, appropriately designed studies are needed.

## Introduction

The prevalence of diabetes is increasing globally, and management of diabetic complications is particularly important. [1], [2], [3] Diabetic nephropathy, resulting in end-stage renal events requiring renal replacement therapy, is one of the most common complications. Furthermore, in the course of diabetic nephropathy, patients have higher rates of mortality from cardiovascular disease. [4] Albuminuria is an early marker of diabetic nephropathy, and previous reports described the association between albuminuria and risks of adverse cardiovascular and kidney events. [5], [6] Albuminuria is often used as a surrogate marker for the risk of fatal and non-fatal events in clinical trials of antihyperglycemic medications or in antihypertensive therapy. [7], [8], [9] Similarly, low eGFR, which is a common manifestation of progressed diabetic nephropathy, has also been demonstrated to be an independent risk factor for cardiovascular events and death. [10], [11] Recent evidence suggests that both high albuminuria and low eGFR are independent risk factors for progressive kidney failure and cardiovascular disease. [10] In addition, the magnitudes of risk for progressive kidney failure, cardiovascular disease, and all-cause mortality were different between studies, and the unevenness may have been due to differences in study design or characteristics of participants. It is important to clarify these problems to apply this evidence to individuals.

To manage diabetic nephropathy, it is necessary to clarify the precise magnitude of the risks for cardiovascular mortality, all-cause mortality, and renal events according to the status of the patient. These observations may be useful for the screening of high-risk patients or considering interventions. Therefore, we conducted a systematic review and meta-analysis of published studies on diabetic nephropathy to provide an accurate estimation of the influence of albuminuria and low eGFR.

## Methods

### Data Sources and Searches

We conducted a systematic review of disease prognosis. A systematic review of the available literature according to MOOSE (meta-analysis of observational studies on epidemiology) guidelines was conducted. MEDLINE (http://ovidsp.ovid.com/), EMBASE (http://www.embase.com/), and CINHAL (http://www.ebscohost.com/cinahl/) from 1950 until December 2010 were searched, and the related literature were identified. Search strategies consisted of medical subject headings and text words, including all spellings of proteinuria, albuminuria, microalbuminuria, macroalbuminuria, and glomerular filtration rate combined with cardiovascular diseases, mortality, renal events (Table 1), and limited to cohort studies of diabetic patients. References from identified studies were also screened manually.

**Table 1:** Search Strategies.

1: diabetes mellitus AND (proteinuria OR albuminuria OR microalbuminuria OR macroalbuminuria)
2: (diabetic nephropathy)
3: (kidney failure, chronic) OR (glomerular filtration rate)
4: (cardiovascular diseases) OR (cerebrovascular disorders)
5: mortality OR death
6: (cohort studies) OR (case-control studies)
(1 or 2) and (3 or 4 or 5) and 6

terms associated with Medical Subject Headings.

### Study Selection

Studies were included if they were cohort studies on diabetic patients that estimated the relative risk (RR) and 95% confidence intervals (CIs) of albuminuria or low eGFR on cardiovascular mortality, all-cause mortality, or renal events, and the estimates were derived from Cox proportional hazard models. The definitions of albuminuria were pre-specified (Table 2). Studies were included if they met the definitions of albuminuria in Table 2. Cardiovascular mortality was defined as death from coronary events and/or stroke, which may be on the basis of International Classification of Diseases codes. Renal events were defined as renal replacement therapy, renal transplantation, or loss of renal function. Loss of renal function is defined as sustained eGFR or creatinine clearance below 60 ml/min/1.73 m^2^ or less, halving of eGFR, or doubling of serum creatinine.

**Table 2:** Definitions of Albuminuria.

Measurement Method	Microalbuminuria	Macroalbuminuria	Any level of albuminuria
24 hour urine collection	30–300 mg/day or 20–200 µg/min	>300 mg/day or >200 µg/min	>30 mg/day or >20 µg/min
(proteinuria)	N/A	>0.3–0.5 g/day	N/A
Spot urine albumin creatinine ratio	30–300 mg/g or 3.4–34 mg/mmol	>300 mg/g or >34 mg/mmol	>30 mg/g or >3.4 mg/mmol
(proteinuria)	N/A	>0.3–0.5 g/g	N/A
Spot urine albumin concentration	3–30 mg/dl	>30 mg/dl	>3 mg/dl
(proteinuria)	N/A	>0.3–0.5 g/l	N/A
Spot urine dipstick	Specific microalbuminuria dipstick positive	N/A	N/A

Abbreviation: N/A, not available.

Based on Sarnak et al. [12].

### Data Extraction and Quality Assessment

The literature search and screening were performed by two of the authors (TT and MS). Authors independently judged the contents of abstracts and full papers in duplicate using standardized data collection form. Additional data were not collected from authors of literature. To eliminate the potential influences of specific disease, studies were excluded if their cohorts included patients with specific complications. Studies were also excluded if they reported estimates of influences without any information about standard error, and if they did not yield an estimate that was not adjusted at least by age.

### Data Synthesis and Analysis

Random-effects model were used to obtain summary estimates of RR and 95% CI. Summary estimates were obtained separately according to the level of albuminuria (microalbuminuria, macroalbuminuria, any level of albuminuria). If only subgroups of the estimate were reported (e.g., by gender), these were pooled by fixed-effects model as a within-study summary estimate. We also investigated studies providing RR associated with low eGFR according to the level of albuminuria. If the study population was representative of a particular level of eGFR (e.g., eGFR >60), it was handled as stratified. To evaluate the influences of albuminuria and low eGFR, compare the relative risks pooled by fixed-effects model according to stratified category of albuminuria (micro- and macroalbuminuria), low eGFR (< 60 mL/min/1.73 m^2^) and normal eGFR (≥ 60 mL/min/1.73 m^2^) regardless of the reference category of eGFR. Heterogeneity between studies was assessed using Cochran Q test and I^2^ value. Potential sources of heterogeneity were examined by subgroup analysis comparing summary estimates from subset of studies categorized by characters of participants or study design. Univariate meta-regression was used to compare the subgroups. Begg’s test [13] and Egger’s test [14] were used to evaluate possible publication bias (where *P*<0.05 was taken to indicate statistical significance). To evaluate an influence of a single study, sensitivity analysis is performed to examine the exclusion of any single study altered the magnitude of relative risk or test for heterogeneity. All analyses were performed using Stata (release 11.2; Stata Corporation, College Station, TX). For all tests, a two-sided *p*-value below 0.05 was considered significant.

## Results

### Literature Search and Characteristics of Studies

**Figure 1:**
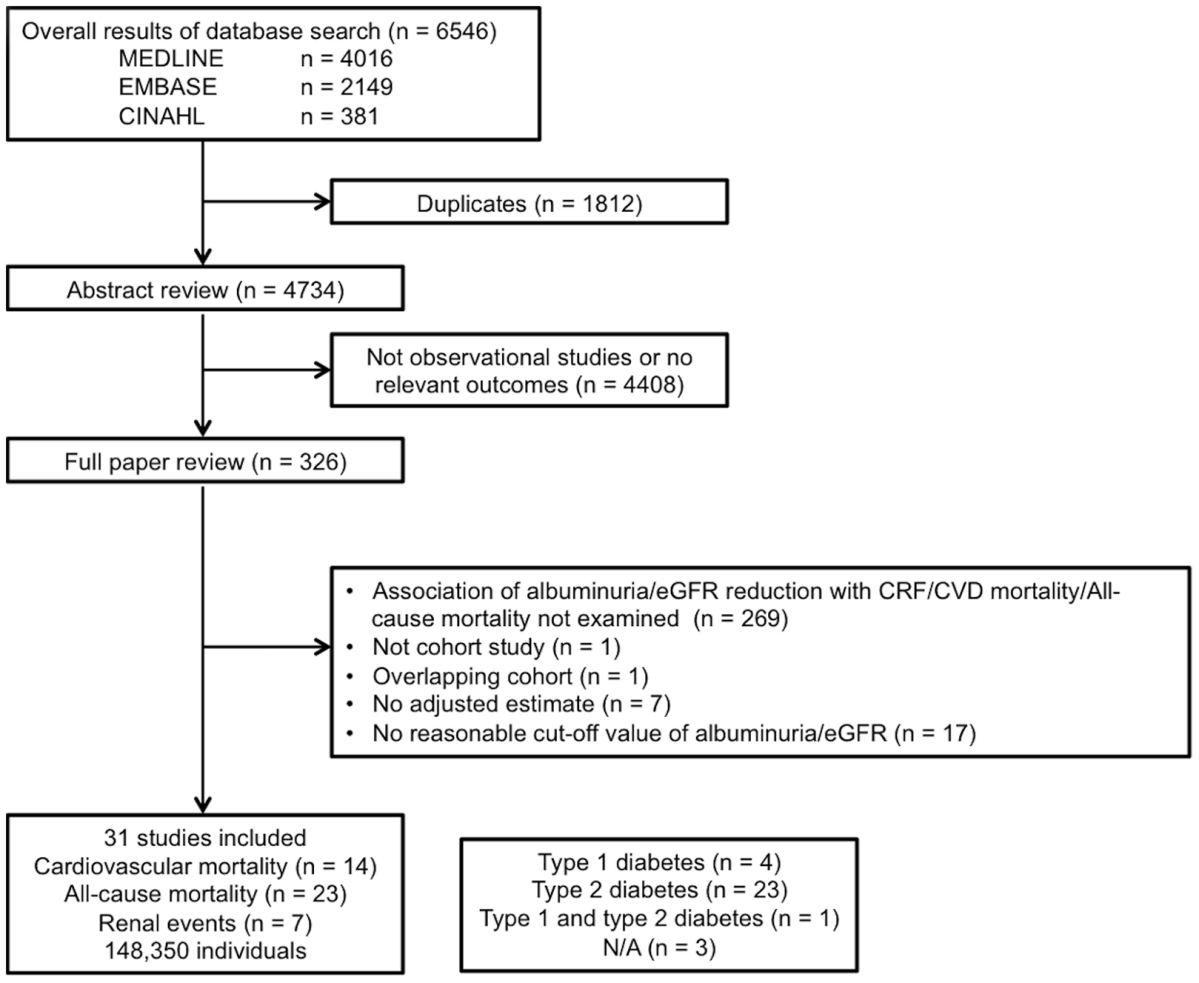
Process for identification of eligible studies Abbreviation: N/A, not available.

The systematic database search yielded 6546 studies, of which 326 papers were reviewed in full (Figure 1). Finally, 31 studies that fulfilled the criteria were included in the analysis, including information for 148350 participants. The crude incidence rates were 19.1 deaths from cardiovascular disease, 35.7 deaths, and 11.7 renal events (per 1000 person-years, respectively). The process of study identification is shown in the flow chart, and the study characteristics are listed in Table 3 and Table 4. Studies consisted of four studies of type 1 diabetic patients, 23 studies of type 2 diabetic patients, one study of type 1 and type 2 diabetic patients, and 3 studies of unknown type of diabetic patients. The study size was in the range of 146 to 94934, and the average follow-up period was in the range of 3 to 19 years. Regarding cardiovascular mortality, Asian population study was not included according to the criteria. We pooled the risk of two studies [15], [16] reporting only subgroups of the estimate.

**Table 3:** Characteristic of Studies Reporting on the Association between Albuminuria or low eGFR and Subsequent Risk of Adverse Outcomes.

Author	Year	Country	Study size	%male	%white		Endpoints^a^		No. of CV mortality	No. of all-cause mortality	No. of renal events
Jager[15]	2010	Netherlands	173	48.0	100.0	CV mortality			16		
O'Hare[16]	2010	US	94,934	98.0	87.0		All-cause mortality			25481	
Grauslund[17]	2010	Denmark	389	55.0	N/A	CV mortality	All-cause mortality		N/A	117	
Molitch[18]	2010	US	1,439	52.5	N/A			Renal events			89
Ninomiya[10]	2009	Multicountries	10,640	57.0	N/A	CV mortality	All-cause mortality	Renal events	432	817	107
Groop[19]	2009	Finland	4,201	51.8	N/A		All-cause mortality			291	
de Boer[20]	2009	US	691	42.1	80.6	CV mortality	All-cause mortality		169	378	
Vlek[6]	2008	Netherlands	759	76.5	N/A	CV mortality	All-cause mortality		49	82	
Luk[21]	2008	China	5,829	49.8	N/A			Renal events			741
Tong[22]	2007	China	4,416	42.9	N/A		All-cause mortality	Renal events		110	221
Bruno[23]	2007	Italy	1,538	43.4	N/A	CV mortality	All-cause mortality		331	670	
Roy[24]	2006	US	725	41.7	0.0		All-cause mortality			131	
So[25]	2006	Hong Kong	4,421	43.2	N/A			Renal events			212
Retnakaran[26]	2006	UK	5,032	59.0	81.0			Renal events			584
Xu[27]	2005	USA	1,953	37.6	N/A^e^	CV mortality	All-cause mortality		223	627	
Yuyun[28]	2003	UK	427	62.1	N/A		All-cause mortality			56	
Bruno[29]	2003	Italy	1,408	43.6	N/A			Renal events			82
Jude[30]	2002	UK	340	66.5	66.8	CV mortality	All-cause mortality		44	63	
Ostgren[31]	2002	Sweden	400	50.5	N/A		All-cause mortality			131	
Stehouwer[32]	2002	Netherlands	328	61.6	N/A		All-cause mortality			113	
Gerstein[33]	2001	North and South America and Europe	3,498	62.9	N/A		All-cause mortality			431	
de Grauw[34]	2001	Netherlands	262	39.0	N/A		All-cause mortality			57	
Florkowski[35]	2001	New Zealand	447	46.5	N/A		All-cause mortality			187	
Casiglia[36]	2000	Italy	683	50.2	N/A	CV mortality			68		
Valmadrid[37]	2000	US	840	45.0	N/A	CV mortality	All-cause mortality		364	529	
Hänninen[38]	1999	Finland	252	53.2	N/A		All-cause mortality			21	
Mattock[39]	1998	U.K.	146	56.2	100.0	CV mortality	All-cause mortality		20	36	
Beilin[40]	1996	Australia	666	47.1	N/A	CV mortality	All-cause mortality		80	167	
Rossing[41]	1996	Denmark	939	52.5	N/A	CV mortality	All-cause mortality		74	207	
Gall[42]	1995	Denmark	328	61.5	N/A	CV mortality			29		
Neil[43]	1993	U.K.	246	50.8	N/A		All-cause mortality			93	

Follow-Up (years)	Mean age (years)	Type of DM^b^	Duration of DM (years)	mean eGFR (ml/min/1.73m^2^)	sBP (mmHg)	dBP (mmHg)	Study type^c^	Use of RASS inhibitors (%)	Adjustment of BP or HT	Stratification of eGFR (ml/min/1.73m^2^ )	Level of Adjustment^d^
	64	T2DM	N/A	67.8	139	83	Obs	N/A	NO	N/A	Age, sex, obesity, HT, TCHOL, TG, HDL, preexistent IHD, current smoking
6.4	66.6	N/A	N/A	75.3	139	74	Obs	60.7	YES	≥90, 89-60, 59-45, 44-30, 29-15	Age, sex, BMI, sBP, dBP, race, eGFR, comorbidity, medication use
13	45.8	T1DM	30.0	68.0	N/A	N/A	Obs	N/A	NO	N/A	Age, sex, DM duration
19.3	27.1	T1DM	5.8	112.6	114	72	Trial	51.0	NO	N/A	Mean arterial pressure, ACE inhibitor use
4.3	66	T2DM	7	80.6	145	81	Trial	50.0	YES	eGFR ≥90/60-89/<60	Age, sex, DM duration, HbA1c, BMI, sBP, history of currently treated HT, logTG, HDL, LDL, ECG abnormalities, current smoking, current drinking, history of macrovascular dis.
7	33.0	T1DM	23.2	75.9	134	80	Obs	28.9	NO	N/A	Age, duration of DM, HbA1c, eGFR, macrovascular disease
10	77.7	N/A	N/A	76.0	138	69	Obs	25.3	YES	60 ≥, <60	Age, sex, BMI, HT, DM duration, TCHO, hypoglycemic medications, race, smoking, lipid-lowering medications, prevalent cardiovascular disease, and prevalent congestive heart failure.
4	59.7	N/A	N/A	113.3	142	82	Obs	22.4	YES	>60, <60	Age, sex, sBP, dBP, HDL, LDL, vascular history, smoking
4.6	54.1	T2DM	6.2	91.0	133	76	Obs	N/A	YES	N/A	Age, sex, BMI, HT, DM duration, HbA1c, Retinopathy, central obesity, hypertriglyceridemia
3.4	57.6	T2DM	5.3	110.3	135	77	Obs	9.3	YES	N/A	Renal failure: Age, waist circumference, sBP; All-cause mortality: Age, sex, BMI
11	68.6	T2DM	10.8	N/A	155	78	Obs	N/A	YES	>60, <60	Age, sex, HT, HbA1c, apoB/apoA1, smoking, fibrinogen
3	29	T1DM	8.0	N/A	N/A	N/A	Obs	N/A	YES	N/A	Age, BMI, dBP, diabetic retinopathy severity level, socio economic status, macroangiopathy, heavy alcohol consumption
3.3	57.6	T2DM	6.9	91.0	134	77	Obs	37.8	YES	>90/60-89/30-39/15-29	Age, sex, DM duration, HbA1c, BMI, sBP, retinopathy, TG, HDL, LDL, smoking, RAAS inhibition
15	52.4	T2DM	0	82.3	135	83	Trial	N/A	YES	N/A	Age, sex, ethnicity, Cr, smoking, waist, height, sBP, retinopathy
8.8	57.1	T2DM	9.7	65.7	136	N/A	Obs	N/A	YES	N/A	Age, sex, BMI, HT, DM duration, TG, HDL, LDL, study center, percent of American Indian heritage, current alcohol drinking, smoking, preexisting CVD
5	53.4	T1+T2DM	14.3	N/A	N/A	N/A	Obs	N/A	YES	N/A	Age, sex, BMI, mean BP, duration of DM, TCHO, smoking, type of DM, baseline cardiovascular history, rate of change of albuminuria over 1 year
6.7	68.1	T2DM	10.7	66.0	154	88	Obs	N/A	NO	N/A	Age, sex, attained time of follow-up
5.3	60.8	T2DM	5.1	N/A	161	88	Obs	N/A	YES		Age, sex, HT, DM duration, HbA1c, TCHO, sCr, race, pre-existing IHD
5.9	69.6	T2DM	8.9	89.0	160	84	Obs	N/A	NO	N/A	Age and sex
9.0	53.8	T2DM	6.4	67.6	151	86	Obs	N/A	YES	N/A	Age, sex, BMI, sBP, DM duration, HbA1c, TCHOL, prior cardiovascular disease
4.5	65.4	T2DM	11.4	73.5	142	80	Trial	50.5	YES	N/A	Age, sex, abdominal obesity, HT, DM duration,HbA1c, dyslipidemia, diabetes status, smoking status, and sCr, use of oral agents or insulin
6	66	T2DM	5	N/A	155	82	Obs	N/A	NO		Age, sex, duration of DM
10	62.2	T2DM	9.6	N/A	N/A	N/A	Obs	N/A	YES	N/A	Age, sex, DM duration, BMI, HbA1c, HT, PVD, smoking, glucose, TCHOL, HDL, TG, cerebrovascular disease, peripheral neuropathy, coronary artery disease, metformin, sulphonylurea, combined oral, insulin
6	63.2	T2DM	N/A	N/A	156	90	Obs	N/A	YES	mean -2S.D. >60	Age, coronary artery disease, sustained arterial hypertension
12	67.9	T2DM	15.1	N/A	143	76	Obs	N/A	YES	N/A	Age, sex, glycemic control, insulin use, alcohol intake, physical activity, history of CVD, intake of antihyptertensive agents, and the presence and severity of diabetic retinopathy
5	58	T2DM	6.0	N/A	145	90	Obs	N/A	NO	N/A	Age, sex, coronary heart disease
7	59	T2DM	5	N/A	144	84	Obs	N/A	NO	N/A	Age, sex, HbA1c, TCHO, preexistent coronary heart disease
4.8	63	T2DM	13	N/A	154	82	Obs	N/A	YES	N/A	Age, sex, DM duration, HbA1c, BMI, sBP, dBP, retiinopathy, TCHO, HDL, TG, age at diagnosis, smoking, fasting glucose, urea, loss of pinprick sensation, leg claudication, number of abscent foot pulses, CHD, carebrovascular disease
9.2	39.6	T2DM	18.6	N/A	136	82	Obs	N/A	YES	N/A	Age, sex, HT, smoking, HbA1c, smoking, height, sCr, social class, overt nephropthy
5.3	55.9	T2DM	6.3	N/A	151	86	Obs	N/A	YES	N/A	Age, HbA1c, sBP, coronary heart disease
6.1	66.2	T2DM	9.1	N/A	160	89	Obs	N/A	NO	N/A	Age, DM duration, retinopathy, lens opacity, intermittent claudication

a Endpoints: CV mortality, cardiovascular mortality.

b Type of DM: N/A, type of DM is not documented; T1DM, population with type 1 DM; T2DM, population with type 2 DM.

c Study type: Obs, based on the cohort of observational study; Trial, based on the cohort of clinical trial.

d Level of Adjustment: ACE, angiotensin converting enzyme; Apo, apolipoprotein; BMI, body mass index; CVD, cardiovascular disease; dBP, diastolic blood pressure; DM, diabetes mellitus; ECG, electrocardiogram; HbA1c, glycosylated hemoglobin A1c; HDL, high-density lipoproteins; HT, hypertension; IHD, ischemic heart disease; LDL, low-density lipoproteins; PVD, peripheral vascular disease; RAAS, Renin-Angiotensin-Aldosterone System; sBP, systolic blood pressure; sCr, serum creatinine TCHO, total cholesterol; TG, triglycerides;

e Cohort of American Indians.

Other abbreviations: N/A, not available; CV mortality, cardiovascular mortality; sBP, systolic blood pressure; dBP, diastolic blood pressure.

**Table 4:** Definitions of Albuminuria, eGFR categories and Outcomes.

Author	Urine measurement method^a^	Definition of microalbuminuria	Definition of macroalbuminuria	Definition of any level of albuminuria	eGFR categories	Criteria of renal failure	Criteria of CV mortality	Definition of CV disease^b^
Jager [15]	ACR			>2.0 mg/mmol			ICD code 390–459	Heart/Brain
O’Hare [16]	ACR	30–299 mg/gCr	≥300 mg/gCr					
Grauslund [17]	spot	30–299 mg/L	≥300 mg/L				ICD-9 codes 430.0–438.9ICD-10 codes I20.0–I25.9, I60.0–I60.9	Heart/Brain
Molitch [18]	AER	30–300 mg/24 h	>300 mg/24 h			sustained eGFR<60		
Ninomiya [10]	ACR	30–300 mg/gCr	>300 mg/gCr		>90, 60–89, <60	death as a result of kidney disease, requirement for dialysis or transplantation, or doubling of serum creatinine to >200 µmol/L	death as a result of coronary heart disease or cerebrovascular disease	Heart/Brain
Groop [19]	AER	20–200 µg/min	>200 µg/min					
de Boer [20]	ACR			≥30 mg/gCr	≥60, <60		death from coronary heart disease, myocardial infarction, sudden cardiac death, or stroke	Heart/Brain
Vlek [6]	ACR			>3 mg/mmol	>60, ≤60		Vascular death, Stroke, Myocardial infarction	Heart/Brain
Luk [21]	ACR	2.5–30 mg/mmol (women) 3.5–30 mg/mmol (men)	>30 mg/mmol			ICD-9 code 250.4, 585, 586 ICD-9 procedure code 39.95 (hemodialysis), 54.98 (peritoneal dialysis)		
Tong [22]	ACR	3.5–25 mg/mmol	≥25 mg/mmol			eGFR halving, eGFR <15 ml/min/1.73 m^2^, death as a result of renal causes or need for dialysis		
Bruno [23]	AER	20–200 µg/min	>200 µg/min		≥60, <60		ICD code 390–459	Heart/Brain
Roy [24]	AER	20–200 µg/min	>200 µg/min					
So [25]	ACR	3.5–25 mg/mmol	≥25 mg/mmol	>3.5 mg/mmol	>90, 60–89, 30–59, 15–29	Reduction in eGFR by 50% or progression to eGFR 15 ml/min/1.73 m^2^ (stage 5) or renal dialysis or death secondary to renal causes		
Retnakaran [26]	spot	50–299 mg/L	≥300 mg/L			Creatinine clearance ≤60 ml/min per 1.73 m^2^		
Xu [27]	ACR	≥30, <300 mg/gCr	≥300 mg/gCr				definite fatal MI, definite sudden death due to CHD, definite or possible fatal CHD, definite or possible fatal stroke, definite or possible fatal CHF, and other fatal CVD	Heart/Brain
Yuyun [28]	AER	30–300 mg/24 h	>300 mg/24 h					
Bruno [29]	AER	20–200 ug/min	>200 ug/min			ESRD (need for dialysis) or chronic renal failure		
Jude [30]	PER		Urine protein ≥0.5 g/24 h				from death certificates	Heart/Brain
Ostgren [31]	qualitative	Specific microalbumiuria dipstick positive						
Stehouwer [32]	AER	30–299 mg/24 h	≥300 mg/24 h					
Gerstein [33]	ACR			>2.0 mg/mmol exclude dipstick–positive proteinuria				
de Grauw [34]	spot	20–200 mg/L	>200 mg/L					
Florkowski [35]	spot			≥50 mg/l				
Casiglia [36]	AER	30–300 mg/24 h	>300 mg/24 h		>60, ≤60		from the hospital of physicians’ files	Heart/Brain
Valmadrid [37]	qualitative	Agglutination inhibition assay positive,and reagent strip negative	Urine protein ≥0.3 g/L				ICD9 codes 402, 404, 410–414, 428, 430–438	Heart/Brain
Hänninen [38]	AER			≥20 µg/min				
Mattock [39]	AER	20–200 µg/min	UAER >200 µg/min				from death certificates	Heart
Beilin [40]	spot	30–300 mg/L	≥300 mg/L				ICD9 codes 390 to 458, 410 to 414	Heart/Brain
Rossing [41]	AER	31–299 mg/24 h	≥300 mg/24 h				from death certificate	Heart/Brain
Gall [42]	AER	30–299 mg/24 h	AER ≥300 mg/24 h				from death certificates	Heart/Brain
Neil [43]	spot	40–200 mg/L	UAC >200 mg/L					

a Urine measurement method: ACR, albumin creatinine ratio; AER, albumin excretion rate; PER, protein excretion rate; spot, spot urinary albumin concentration; qualitative, qualitative detection of albumin in urine.

b Definition of CV disease: Heart, ischemic heart disease; Brain, cerebrovascular disease.

Micro- and macroalbuminuria were defined as risk factors in 25 studies. Any level of albuminuria (i.e., micro- or macroalbuminuria) was defined as a risk factor in 7 studies. In these studies, various means of expression of albuminuria were adopted. The magnitude of microalbuminuria was expressed as urinary albumin excretion rate (*n*?12), urinary albumin-creatinine ratio on spot urine samples (*n*?10), spot urinary albumin concentration (*n*?6), qualitative test of albuminuria (*n*?2), or urinary protein excretion rate (*n*?1). Almost all of the estimates were adjusted for multiple risk factors including age. In one study [17], the estimate was not adjusted for age because age was not a statistically significant risk.

### Association of Albuminuria with Risk of Cardiovascular Mortality

**Figure 2:**
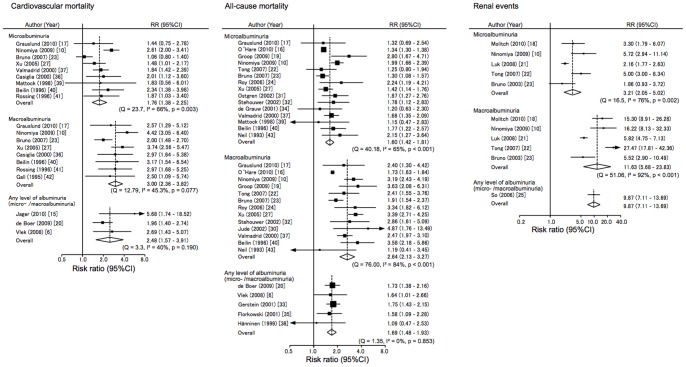
Risk ratio for the association between albuminuria and cardiovascular mortality, all-cause mortality, and renal events compared with normoalbuminuria. Abbreviations: CI, confidence interval; RR, risk ratio.

Microalbuminuria was associated with 1.76 (95% confidence interval [CI] 1.38–2.25) times greater risk of cardiovascular mortality as compared with normoalbuminuria (Figure 2), with strong heterogeneity among studies (I^2^ = 66%, *p* = 0.003 for heterogeneity). We found no significant evidence of publication bias. Subgroup analysis did not determine the suspected source of heterogeneity (Figure S1). Age stratified analysis showed no trends neither micro- nor macroalbuminuria (Figure S2). Macroalbuminuria was associated with about 2.96 (95%CI 2.44–3.60) times greater risk of cardiovascular mortality compared with normoalbuminuria, and there was no significant evidence of heterogeneity among studies. These findings suggest that there is a dose-dependent association between albuminuria and the risk of cardiovascular mortality: the influence of macroalbuminuria was significantly higher than that of microalbuminuria (*p* = 0.026). In the three studies for which information was available, any level of albuminuria was associated with about 2.48 times (95%CI 1.57–3.91) greater risk of cardiovascular mortality compared with normoalbuminuria, without any evidence of heterogeneity in the association.

### Association of Albuminuria with Risk of All-cause Mortality

Summary estimates of the influences of microalbuminuria and macroalbuminuria on all-cause mortality were 1.60 (95%CI 1.42–1.81) and 2.64 (95%CI 2.13–3.27), respectively (Figure 2): the associations were heterogeneous among studies for both (I^2^ = 65% and 84%, both *p*<0.001 for heterogeneity). There was some evidence of publication bias in microalbuminuria and macroalbuminuria (Egger’s test *P*?0.014 and *P*?0.015, respectively), which may have overestimated the strength of the association. Subgroup analysis did not determine the suspected source of heterogeneity. As to the racial difference, relative risks were not significantly different between Asians and non-Asians. A study in veterans (O’Hare et al.) [18] yielded a lower risk of all-cause mortality (HR 1.34 [95%CI 1.30–1.38] for microalbuminuria, HR 1.73 [95%CI 1.63–1.84] for macroalbuminuria), but the source of heterogeneity was not apparent (Figure S1). In age-stratified analysis, there was no significant difference between younger and older age (Figure S2). Sensitivity analysis excluding this study [18], with the highest weight in this meta-analysis, showed a similar relative risk in microalbuminuria (HR 1.65 [95% CI 1.46 – 1.87]) and macroalbuminuria (HR 2.77 [95% CI 2.34 – 3.27]); the test for heterogeneity was insignificant in microalbuminuria (I^2^?41.0%, *P*?0.06), and was still significant for macroalbuminuria (I^2^?51.1%, *P*?0.02). The summary estimate of the influence of any level of albuminuria for the risk of all-cause mortality was 1.69 (95%CI 1.48–1.93).

### Association of Albuminuria with Risk of Renal Events

Summary estimates of the influences of microalbuminuria and macroalbuminuria on renal events were 3.21 (95%CI 2.05–5.02) and 11.63 (95%CI 5.68–23.83), respectively (Figure 2): the risk estimates of micro- and macroalbuminuria were diverse across studies (I^2^ = 76% and 92%, *p* = 0.02 and *p*<0.001 for heterogeneity). We found no significant evidence of publication bias. Subgroup analysis did not show any significant differences between characteristics of participants or study design (Figure S1). Asians have almost the same risk for renal events as non-Asians in both micro- and macroalbuminuria. Age stratified analysis showed no trends in microalbuminuric or macroalbuminuric patients (Figure S2). One study evaluating the influences of any level of albuminuria showed the same trend.

### Combined Impacts of Low eGFR on Albuminuria

**Figure 3:**
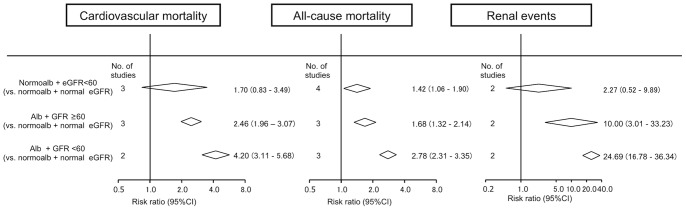
Risk ratio for the association of low eGFR with the risk of each outcome according to the presence of albuminuria, compared with normal eGFR and normoalbuminuria. Albuminuria was defined as any level of albuminuria or pooled estimate of microalbuminuria and macroalbuminuria. Abbreviations: normoalb, normoalbuminuria; alb, albuminuria.

A few studies [6], [10], [19], [20] evaluated the combined influence of low eGFR on albuminuria in terms of the risk for the outcomes. As compared to those with normoalbuminuria, the risk of cardiovascular mortality tended to increase by 1.70-fold (95%CI 0.83–3.49) in subjects with normoalbuminuria and eGFR of <60 mL/min/1.73 m^2^ (Figure 3). Similarly, the presence of albuminuria was significantly associated with 2.46-fold (95%CI 1.96–3.07) increased risk of cardiovascular mortality. Furthermore, subjects with both albuminuria and eGFR <60 mL/min/1.73 m^2^ were at 4.20 times (95%CI 3.11–5.68) higher risk of cardiovascular mortality compared to those with neither of these risk factors.

## Discussion

This study explored the influences of albuminuria and low eGFR on cardiovascular mortality, all-cause mortality, and renal events in diabetic patients using meta-analysis methods with 148350 cases. Microalbuminuria and macroalbuminuria are significant risk factors for each outcome. Similar to the influences of albuminuria, low eGFR also increased the risk of each adverse outcome.

This meta-analysis suggested that low eGFR and albuminuria may be independent risk factors for cardiovascular mortality, all-cause mortality, and renal events. Recent published new CKD staging from Kidney Disease: Improving Global Outcomes (KDIGO) was defined by these two factors, eGFR and albuminuria. [44], [45] However, conventional staging of diabetic nephropathy was classified only by degree of albuminuria. [46] Many reports and meta-analysis indicated albuminuria as one of the main risk factors for cardiovascular mortality and all-cause mortality in diabetic patients. [47] Although the number of reports was limited, some indicated the influences of low eGFR on the risk of each outcome in diabetic nephropathy. [10], [19], [20] However, other reports concluded that low eGFR was not always a significant risk factor for these outcomes. [6], [25] Thus, the influences of albuminuria and low eGFR are not consistent among studies adjusted for each other. Further large prospective studies are needed to clarify the independent influences of albuminuria and low eGFR on the three outcomes in diabetic nephropathy.

The interaction between eGFR and albuminuria may be important in considering the possibility of albuminuria and low eGFR as independent risk factors for the three outcomes. Previous meta-analyses of general and high-risk cohorts indicated no interaction between eGFR and albuminuria on the risks of cardiovascular mortality, all-cause mortality, and renal events. [48], [49] Similarly, in our results of diabetic nephropathy consisting of 4 data or less, stratified analysis demonstrated that the magnitudes of relative risks of these events with low eGFR and albuminuria were almost equivalent to those obtained by multiplying each risk rate of low eGFR and albuminuria. These results suggested that there is no interaction between eGFR and albuminuria in each adverse outcome. In our meta-analysis, only two studies evaluated the interaction between eGFR and albuminuria. [10], [25] One of these studies that included stratified analysis indicated that increasing risk of cardiovascular mortality and all-cause mortality in low eGFR were significantly higher in patients with macroalbuminuria but not those with normoalbuminuria. [25] Moreover, in a previous meta-analysis, one of eight general and high-risk cohorts showed significant interaction between eGFR and albuminuria for the risk of ESRD. [49] Based on these studies, the significance of the interaction between eGFR and albuminuria is still variable. Detailed analysis of cohort studies, including an unusual case of diabetic nephropathy, such as low eGFR with normoalbuminuria and high GFR with macroalbuminuria, are needed to resolve the precise interaction of them.

There was heterogeneity among studies for cardiovascular mortality, all-cause mortality, and renal events in the presence of microalbuminuria or macroalbuminuria. There are some possible causes of the heterogeneity in this study. One of the possible reasons is a large cohort with different results from the others. Another possible reason is the diversity of study design. A large study with an exceptional setting [18] may lead to heterogeneity of the outcome. The report by O’Hare et al. had the highest weight in this meta-analysis, and its relative risk was even lower than the pooled risk of all-cause mortality. [18] Therefore, this large cohort study of veterans should have some different setting from other studies. The multiplicity of study design is an unavoidable limitation of meta-analyses, which is another possible reason of heterogeneity. The entry criteria, treatment, or adjustment for confounders were different between studies, and the different settings may affect results to uneven extents. Although some other factors, such as blood pressure control or use of ACE inhibitors for renal events, are possible factors for heterogeneity, these factors were not fully evaluated in the studies included in this analysis. [50], [51] Based on these results, standardization of study design is needed, including treatment strategy or adjustment of confounders.

As diabetes is a common disease with high risk of macrovascular and microvascular complications, we focused on diabetic patients. In this sense, we excluded patients without diabetes from this study. Due to this restriction of subjects, our study precisely compared the outcomes of the studies of diabetic cohorts. On the other hand, out study was not able to describe the risk of patients with diabetes compared to those without diabetes.

The strength of this study is the listing of all studies allowing readers to see the inconsistency across cohorts. The limitations of this study should also be noted. First, the numbers of studies regarding the associations between low eGFR and cardiovascular mortality, all-cause mortality, and renal events were small. Although low eGFR was considered as a risk factor for cardiovascular events according to the guidelines developed by KDIGO in 2002, there were few studies from this viewpoint prior to this time. [44] Second, each study had its own definition of normal eGFR as the reference category for multivariate analysis. Some studies [10], [19] defined normal eGFR as >90 mL/min/1.73 m^2^, while others [6], [20] used a definition of >60 mL/min/1.73 m^2^. The difference in definition may have affected the magnitude of pooled risk ratio for each outcome. Third, there were differences in measurement and expression of albuminuria, such as daily excretion of albumin, or the ratio of urinary albumin to creatinine. Moreover, measurement of urinary albumin was still not standardized. [52], [53], [54] A standardized method for measurement of albuminuria is essential for comparing data across studies. Furthermore, collection of urine was also not standardized. Spot urine sample collection in the morning or daily collection of urine would lead to different magnitudes of risk ratio.^,^ [55] With regard to expression of urinary albumin, some guidelines [56], [57], [58] use albumin/creatinine ratio. However, other expressions were also used in different studies, such as 24-h excretion or concentration of urinary albumin. Fourth, there may be problems associated with reporting bias, especially for renal events. Some studies measuring serum creatinine at baseline did not report renal outcome. The outcome reporting bias may have increased the influence of renal outcome, which is a very large risk ratio compared with cardiovascular or all-cause mortality. Fifth, the numbers of studies reporting the influence of low eGFR were small. Our search strategy limited objects as “diabetes with albuminuria/proteinuria” or “diabetic nephropathy.” Therefore, studies of diabetic patients with low eGFR may not have been included in our systematic review due to our search strategy. Sixth, making the best use of information about study design or baseline characteristics, the threshold of study size was not used as a limitation in study selection. These selection criteria resulted in more than half of the selected studies consisted of less than 1000 participants.

With regard to the effects of albuminuria and eGFR in diabetic patients, the Chronic Kidney Disease Prognosis Consortium (CKDPC) reported a precise estimate of risk [59]. In addition, our study provided further information showing the inconsistency of study design or subgroup analysis, and presented pooled risk ratio by category of albuminuria and low eGFR for use in clinical care. Moreover, information about intervention or race (except Caucasian) is limited in both the report of CKDPC and this systematic review.

In summary, we conducted a systematic review and meta-analysis, including 148350 cases, and described the impacts of albuminuria and low eGFR on the risks of cardiovascular mortality, all-cause mortality, and renal events. Micro- and macroalbuminuria were significant risk factors for all three outcomes, and low eGFR and albuminuria may be independent risk factors. There was less evidence exploring the influences of low eGFR as independent risk factor on the outcomes. To evaluate the effects of low eGFR, intervention, or race, including Asian subjects, individual patient data meta-analysis or long-term prospective studies based on individual patient data are needed.

## Supporting Information

Figure S1 **Subgroup analysis for examination of potential sources of heterogeneity in the association between micro- or macroalbuminuria and cardiovascular mortality, all-cause mortality or renal events.**
(TIFF)

Figure S2 **Age stratified analysis for the association between albuminuria and cardiovascular mortality, all-cause mortality, and renal events compared with normoalbuminuria.**
(TIFF)
