# Supplementary figures and images for: The Impacts of Albuminuria and Low eGFR on the Risk of Cardiovascular Death, All-Cause Mortality, and Renal Events in Diabetic Patients: Meta-Analysis
Source: PLoS One. 2013 Aug 30;8(8):e71810. doi: 10.1371/journal.pone.0071810 (PMC3797878; doi:10.1371/journal.pone.0071810)

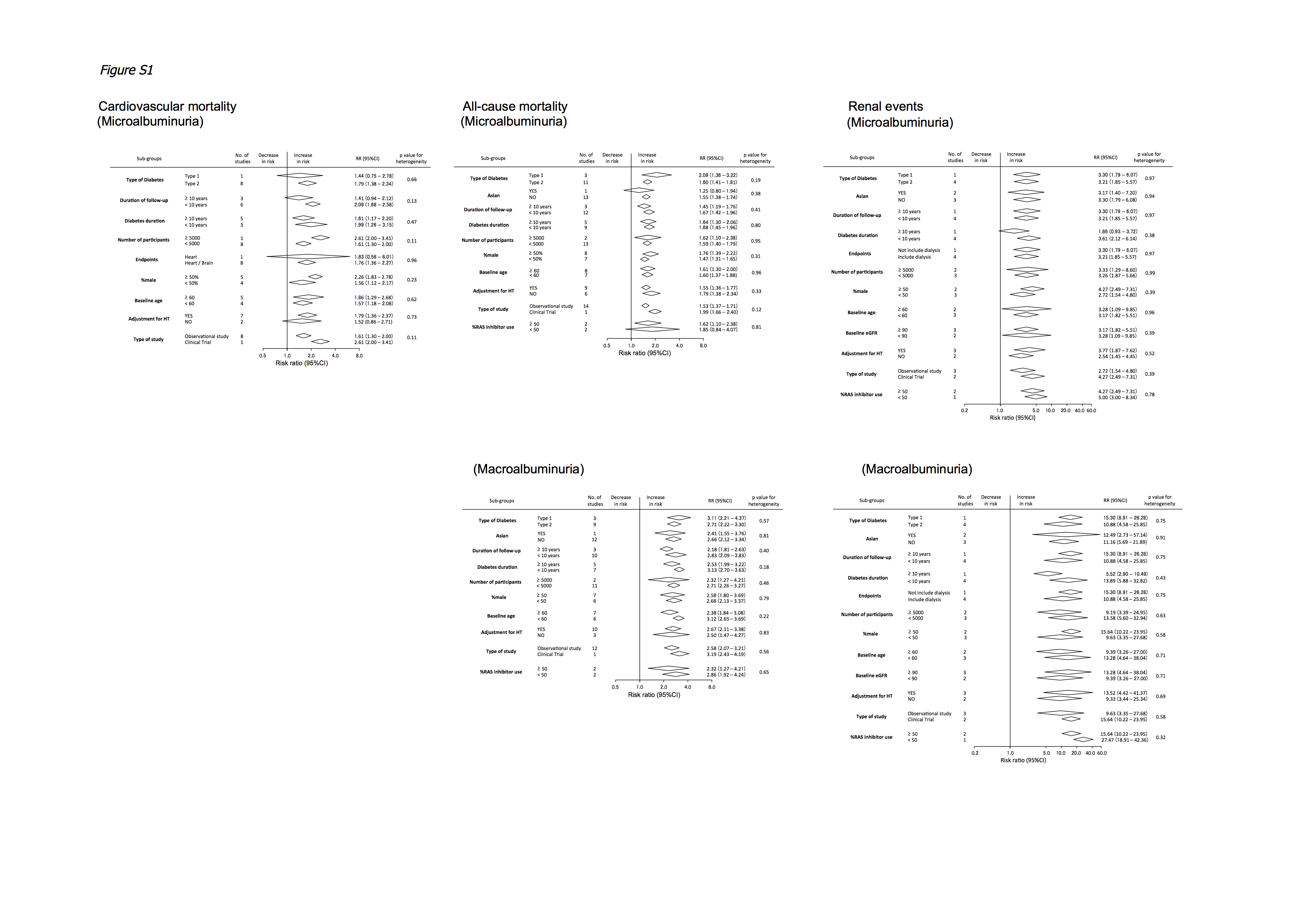

Supplement: Figure S1 — Subgroup analysis for examination of potential sources of heterogeneity in the association between micro- or macroalbuminuria and cardiovascular mortality, all-cause mortality or renal events. (TIFF) [file pone.0071810.s001.tiff]

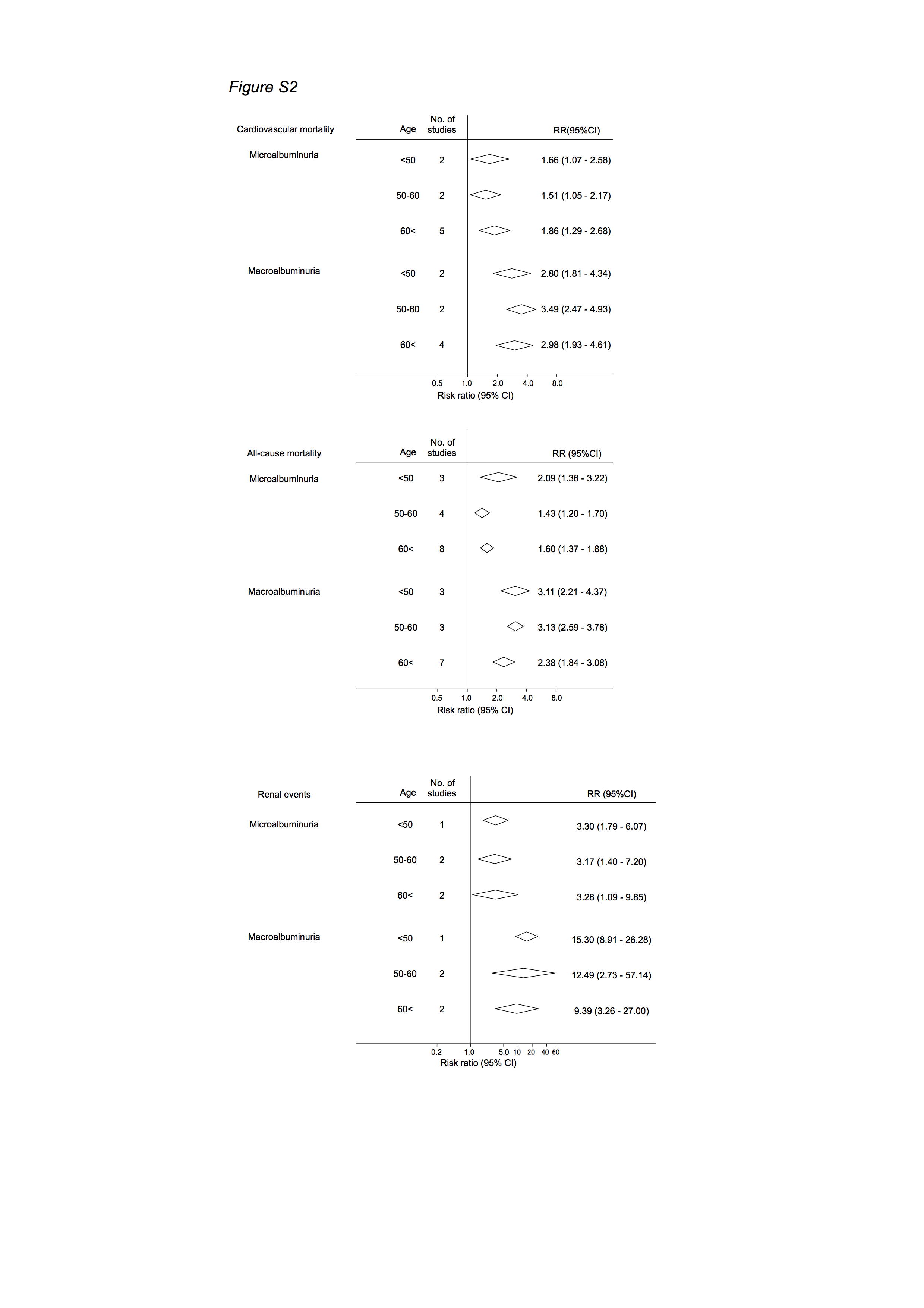

Supplement: Figure S2 — Age stratified analysis for the association between albuminuria and cardiovascular mortality, all-cause mortality, and renal events compared with normoalbuminuria. (TIFF) [file pone.0071810.s002.tiff]
